# Supplementary material for: Bispecific antibodies combine breadth, potency, and avidity of parental antibodies to neutralize sarbecoviruses
Source: iScience. 2023 Mar 31;26(4):106540. doi: 10.1016/j.isci.2023.106540 (PMC10065043; doi:10.1016/j.isci.2023.106540)
Supplement: Document S1. Figures S1–S5 [file mmc1.pdf]

iScience, Volume *26*

Supplemental information

Bispecific antibodies combine breadth, potency, and avidity of parental antibodies to neutralize sarbecoviruses

Laura Radić, Kwinten Sliepen, Victor Yin, Mitch Brinkkemper, Joan Capella-Pujol, Angela I. Schriek, Jonathan L. Torres, Sandhya Bangaru, Judith A. Burger, Meliawati Poniman, Ilja Bontjer, Joey H. Bouhuijs, David Gideonse, Dirk Eggink, Andrew B. Ward, Albert J.R. Heck, Marit J. Van Gils, Rogier W. Sanders, and Janke Schinkel

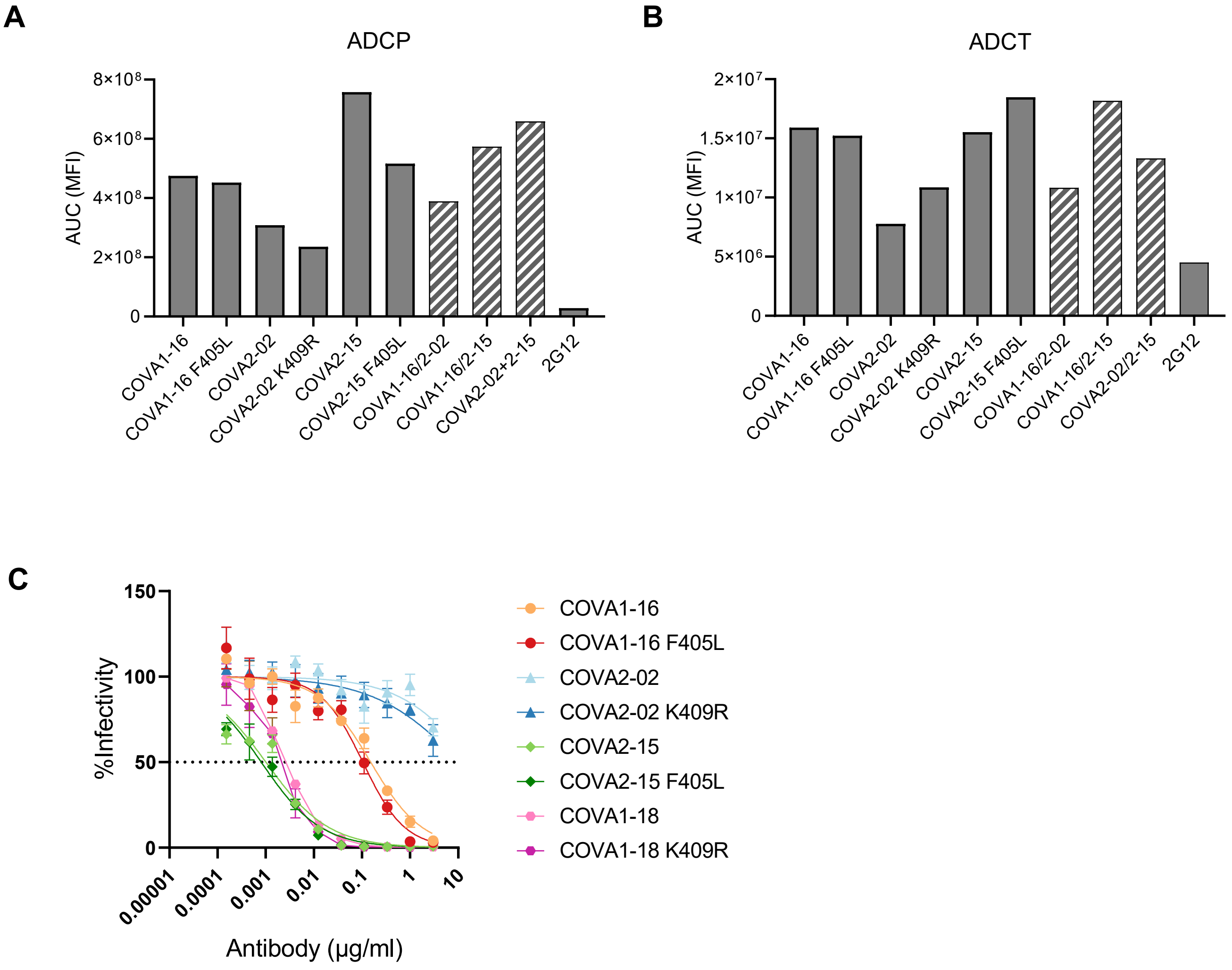

Figure S1. Fc effector functions of bsAbs are retained. Related to Figure 1 and 3. Antibody-dependent cellular phagocytosis (ADCP) (A) and antibody dependent cellular trogocytosis (ADCT) (B) assays were performed and signal was measured using flow cytometry. 2G12, an HIV-1 gp120 specific IgG was used as negative control. Bars represent the area under the curve (AUC) values of the mean fluorescence intensity (MFI). (C) SARS-CoV-2 (Wuhan-Hu-1) pseudovirus neutralization curves of COVA monospecific NAbs and versions containing the F405L and K409R mutations needed for bsAb generation by cFAE. The dotted line indicates 50% infectivity. Curves are representative of two separate experiments performed in triplicate. Data points represent the mean ± SEM.

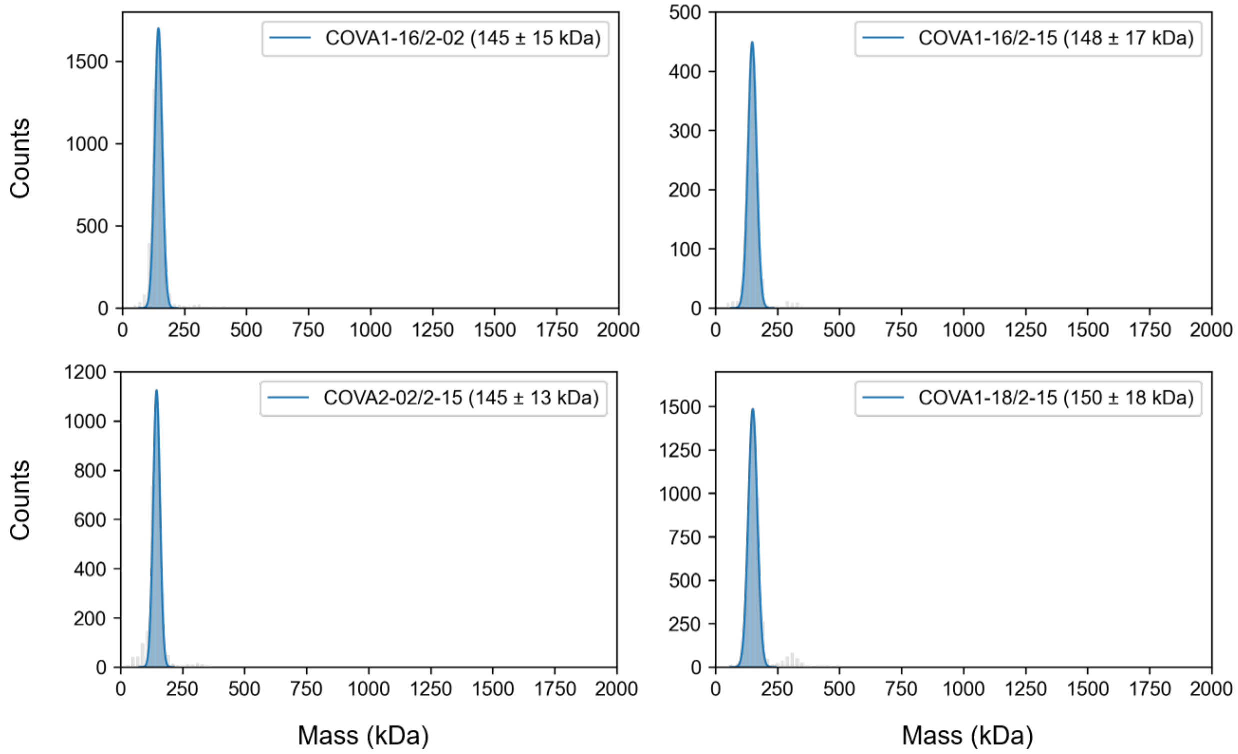

Figure S2. Mass photometry histograms of COVA bsAbs. Related to Figure 2. A single mass distribution at ~150 kDa is observed for all bsAbs, corresponding to the predicted masses of full IgG1s, confirming that the Fab-arm exchange induced formation of bsAbs does not lead to Ab aggregation.

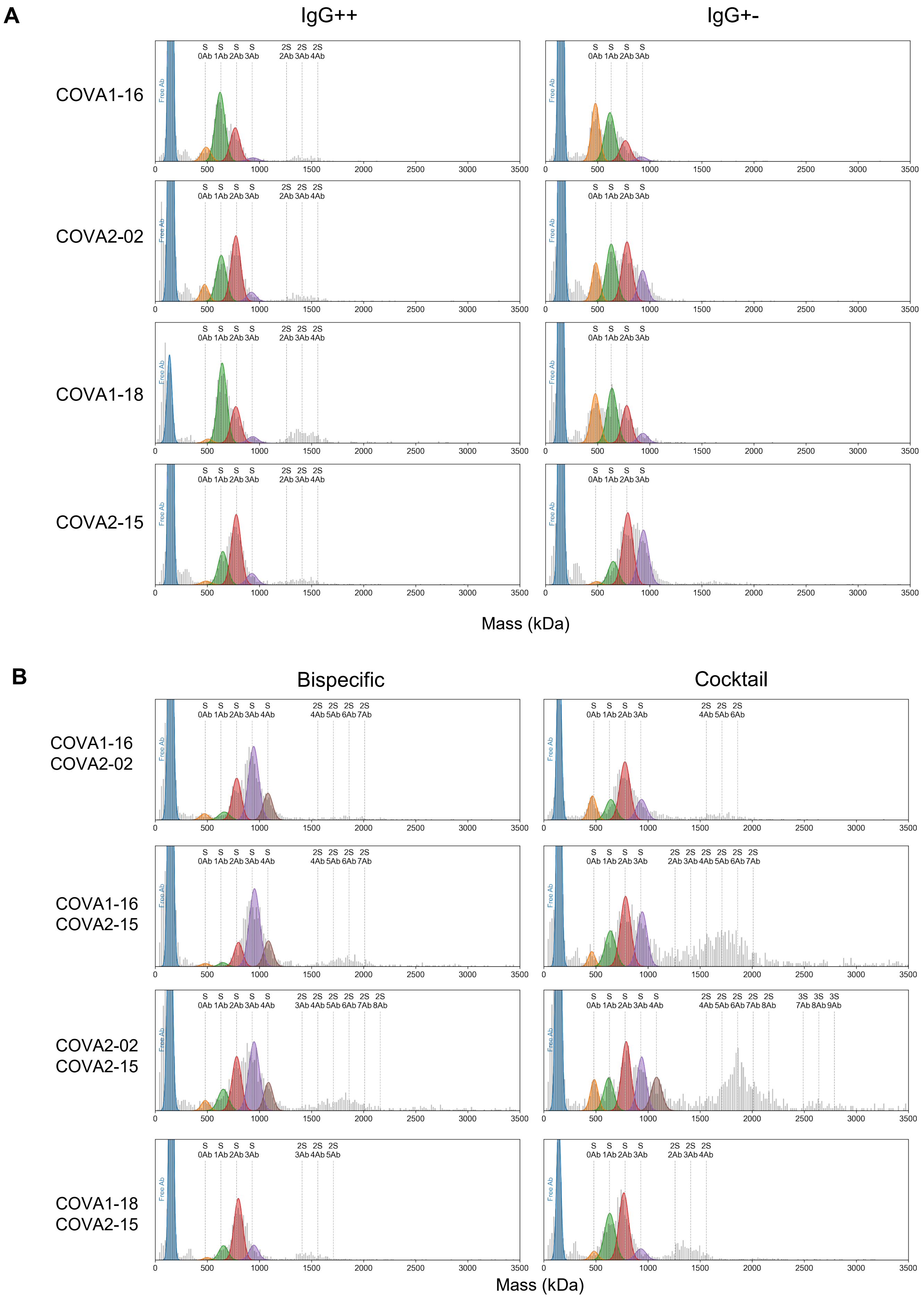

Figure S3. Full MP histograms of COVA monospecific, bispecific and cocktail binding to Wuhan-Hu-1 S. Related to Figure 2. Raw histograms are shown together with the fitted curves for COVA IgG++ and IgG+- constructs (A) and COVA bsAbs and cocktails (B). Besides the complexes of 1-3 Abs to one S trimer, higher stoichiometries such as 2S:2Ab, 2S:3Ab are indicated with dotted vertical lines where applicable.

A

IC50 (μg/mL)

Antibody	Wuhan-Hu-1	Beta	SARS-CoV
COVA1-16	0.08	0.04	0.5
COVA1-16/HC84.26	7.7	2.6	10
COVA2-02	6.5	8	0.3
COVA2-02/HC84.26	10	10	10
COVA2-15	0.002	0.38	10
COVA2-15/HC84.26	0.021	0.42	10
COVA1-18	0.001	10	10
COVA1-18/HC84.26	0.026	10	10

B

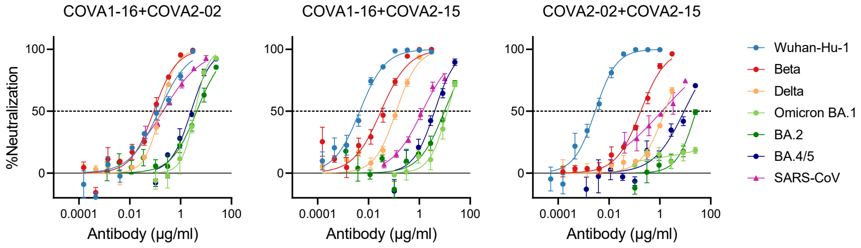

C

IC50 (μg/mL)

	Antibody	Wuhan-Hu-1	D614G	Alpha	Beta	Gamma	Delta	Omicron BA.1	BA.2	BA.4/5	SARS-CoV
Monospecific	COVA1-16	0.08	0.07	0.06	0.04	0.05	0.07	1.7	7.1	2.1	0.55
	COVA2-02	6.5	5	0.5	8	1.2	5	8.9	11.8	9.9	0.3
	COVA2-15	0.002	0.002	0.008	0.4	0.5	1.5	>25	>25	>25	10
Bispecific	COVA1-16/2-02	0.3	0.4	0.1	0.2	0.3	0.2	3.5	9.8	5.6	0.3
	COVA1-16/2-15	0.006	0.006	0.02	0.02	0.05	0.05	17.2	4.4	15.5	3.8
	COVA2-02/2-15	0.007	0.007	0.02	0.2	0.2	0.4	>25	12	18	0.4
Cocktail	COVA1-16+COVA2-02	0.1	0.1	0.08	0.1	0.07	0.2	3.0	5.6	2.3	0.2
	COVA1-16+COVA2-15	0.004	0.003	0.01	0.03	0.06	0.1	9.8	17.4	2.2	1.3
	COVA2-02+COVA2-15	0.004	0.003	0.02	0.3	0.2	1.6	>25	>25	9.5	0.9

Figure S4. Pseudovirus neutralization of SARS-CoV-2 variants and SARS-CoV by COVA monospecific antibodies, bispecific antibodies and cocktails. Related to Figure 3. (A) IC50 values (μg/mL) of COVA monoclonal IgGs in comparison to respective "dead arm" bispecifics. All IC50S greater than 10 μg/mL were rounded up to 10 and were considered non-neutralizing. (B) Representative neutralization curves of 1:1 cocktails of COVA NAbs against SARS-CoV-2 (Wuhan-Hu-1), SARS-CoV-2 variants and SARS-CoV. The dotted lines indicate 0% and 50% neutralization. Data points represent the mean ± SEM of technical triplicates. (C) Summary of all IC50 values of sarbecovirus neutralization by COVA monospecific and bispecific NAbs and corresponding cocktails. Every value represents the mean IC50 of at least two independent experiments performed in triplicate.

A

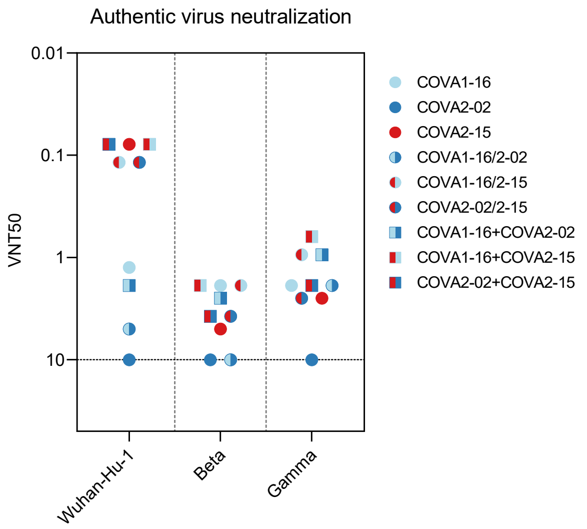

B

		Wuhan-Hu-1		Beta		Gamma	
	Antibody	VNT50	VNT90	VNT50	VNT90	VNT50	VNT90
Monospecific	COVA1-16	1.25	3.75	1.88	5	1.88	3.75
	COVA2-02	10	10	10	10	10	10
	COVA2-15	0.08	0.12	5	10	2.5	10
Bispecific	COVA1-16/2-02	5	10	10	10	1.88	10
	COVA1-16/2-15	0.12	0.47	1.88	2.5	0.94	1.88
	COVA2-02/2-15	0.12	0.47	3.75	5	2.5	3.75
Cocktail	COVA1-16 + COVA2-02	1.88	10	2.5	3.75	0.94	5
	COVA1-16 + COVA2-15	0.08	0.47	1.88	1.88	0.63	1.88
	COVA2-02 + COVA2-15	0.08	0.23	3.75	10	1.88	5

C

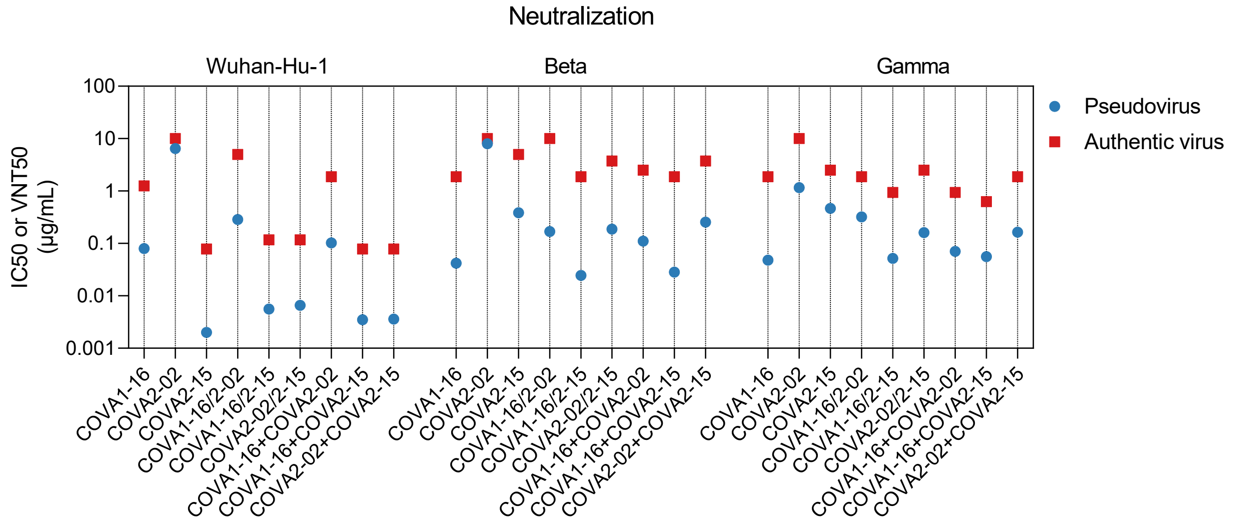

D

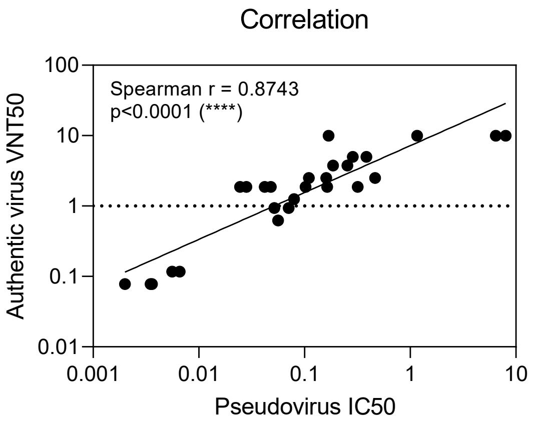

Figure S5. Wuhan-Hu-1, Beta and Gamma authentic virus neutralization. Related to Figure 3. (A) Half-maximal virus neutralization titers (VNT50) of authentic virus neutralization by COVA monospecific (dots of one color) and bispecific (dots of two colors) antibodies and corresponding cocktails of parental NAbs (squares of two colors). Each symbol represents the mean VNT50 value of two replicate measurements. (B) Table with summarized VNT50 and 90% virus neutralization titers (VNT90) of Wuhan-Hu-1, Beta and Gamma neutralization. (C) Comparison of IC50 (pseudovirus neutralization) and VNT50 values (authentic virus neutralization). Each dot represents the mean value from at least 2 separate experiments (IC50 values) or 1 experiment performed in duplo (VNT50 values). (D) Correlation plot of pseudovirus and authentic virus neutralization titers compared for different antibodies and viral strains. Spearman correlation (r) and p value were determined in GraphPad Prism 8.3.0.
